# Review of the Progress in Histology

**Published:** 1882-10

**Authors:** S. E. Davenport

**Affiliations:** New York.


### ARTICLE IV.

### *Review of the Progress in Histology.*

BY DR. S. E. DAVENPORT, OF NEW YORK.

[Read before the Connecticut Valley Dental Society, at Amherst, Mass., June, 1882.]

The chairman of Section 1 desires to thank *all* the members of the section for the very cordial support he has received, and particularly those who have taken any notice of his appeals for aid, even though it may have been with a "not prepared."

The dentist should have a good knowledge of general anatomy, and an accurate understanding of the arrangement of the tissues entering into the formation of, and lying contiguous to, the buccal cavity. In England the student of denistry is required to pass as rigid an examination upon anatomy as is the student of medicine. In this country a full course of dissection has been made compulsory at many of our dental colleges, while at the excellent institution of Harvard and the Dental Department of the University of Pennsylvania, the instruction and requirements are in every way the same, for the dental as for the medical students, in both anatomy and physiology.

In those dental colleges where a course of dissection is not obligatory, many students shrank from taking the same, partly from feelings of disgust and horror at the idea of spending so much time in close proximity to the cadavera. That feeling soon disappears upon the use of the scalpel, and the testimony of all who are really interested in anatomy will probably agree with that of the writer, in calling time at the dissecting table both profitably and pleasantly spent.

(The department of physiology was accepted by a member of the section, but his necessary absence from home for several wesks has prevented the preparation of his report.)

The idea that animals and plants, however complex their structure, are composed of a limited variety of elementary parts, has been held for many centuries. Aristotle referred to it in his writings, while Galen, the father of medical science, went so far as to claim that what we now call tissues, muscle, nerve, bone, cartilage, tendon, etc., were the ultimate elementary parts, or "*partes similares*," as he called them. It would seem strange that Galen, and the anatomists of his time, should claim that in the "*partes similares*" they had arrived at parts no longer analyzable, did we not appreciate the difficulties which beset the path of histologist in those days in the absence of compound microscope. To be sure, we know that simple lenses were known to the Greeks and Romans over twenty centuries ago, but their use was confined to the generation of heat by the refraction of the Sun's rays, and the magnifying possible with the simple lens, until, in 1590, the Jansens of Holland combined the power of two lenses and formed the first compound microscope.

The next attempt at the discovery of ultimate physical element was made by Haller, who in the eighteenth century promulgated the fiber theory, claiming that all tissues were composed of fibres and an interconnecting organized concrete. In the early part of the present century their appeared the writings of a large number of observers who claimed the fiber of Haller was not the ultimate element but that it could be separated into globules, which, they claimed, would form fibers, veins, arteries, etc., by the linear apposition and connection of these ultimate globular elements. All these writers agreed that there was a structureless, semi-fluid, glue-giving, intermediate material of which some tissues, notably cartilage, were for the most part composed.

The cell doctrine, as it is called, had for its founders the two eminent German histologist, Schleiden and Schwann, who in 1838–9 published their views—those of Schleiden being based his study of the minute structure of plants, and Schwann's upon the application of Schleiden conclusions to the histology of the animal tissues. There cannot be too much credit given to Drs. Schleiden and Schwann for their patient labor with the microscope, for the cell theory, although incorrect in its claims as to the presence of cells distinct and separate—the unit upon which all development depends—was a great advance upon all the theories previously promulgated, and was so beautiful when explained and understood as to stamp each student who gave it attention an ardent admirer of histology forever. The writer confesses that he was much grieved when first it was demonstrated to him that the cell theory was not tenable. There was something so satisfactory in the idea of being built up of six or eight different kinds of cells, varying in shape according to the special needs of the locality, and the student imagined he could feel the blood pabulum coursing through his arteries, and when it reached the capillaries, there came the gentle though discernible grasp of the cell nucleolus, which metamorphosed the pabulum into living matters—soon losing its life, however—being changed gradually into nucleolus, cell contents, and then formed material—this last being just outside that ever prent but never seen "cellwall," which has, in the much controversy regarding it, been likened to everything from the skin of an orange to the halo about Cupid's head.

Then, too, if it was ever our misfortune to meet with a person whose good nature was not sufficiently developed to enable him to bear all of our peculiarities, under the cell doctrine we could charitably conclude that in his organization the squamous and polar cells had unfortunately been transposed, or that when he was formed, the usual variety of cells not being obtainable, he had been obliged to close out a job lot of perhaps "*wandering*" cells. Now all is changed. No longer can we liken the human frame to a chimney, built up like it with cells for bricks, entirely separate and individual except for the mortar-like connection afforded by the intermediate formed material.

Let us hope that with the loss of our chimney all *smoke* has also vanished, and that the views held to-day by Carl Heitzmann and his disciples may prove as true through time as to the earnest student they now appear to be.

Carl Heitman was born in the year 1836 at the southern military boundary of Hungary, where his father was on duty as an army surgeon. Inheriting a strong liking for medicine and surgery, he studied these sciences first in Pest, the capital of Huhgary, and Vienna, Austria. In 1859 he graduated with honor at the University of Vienna, and was immediatly elected assistant surgeon to the late eminent Prof. Schulh. In 1862 he became associated with the late Prof. Hebra, who soon published the greatest work on skin diseases in existence—six of the ten volumes being illustrated by Carl Heitzmann.

During the next five years Dr. Heitzmann pursued surgical, anatomical and hisological studies, and illnstrated and issued several works upon surgery and anatomy. His Manual of Surgey, two volnmes, has reach five editions and is translated into several languages, while Anatomy, also two volumes, containing 600 large wood cuts, has reached two editions. His first histological researches were published in 1868, and were upon the minute anatomy of the small intestine.

In 1872, '73 and '74 there appeared in the Transactions of the Imperal Academy of Science, in Vienna, various papers by Carl Heitzmann upon general histology, the new and peculiar claims of which although scoffed at at the time by the originators and supporters of the cell theory, were destined soon to overthrow that theory and to make converts of many who at first were loudest in their jeering. Early in the year 1874 Dr. Heitzmann was elected Lecturer of Morbid Anatomy in the University of Vienna, where he was in the direct line of promotion to the Chair of morbid Antomy, to succeed the late Rokitansky; but on account of the new views upon histology held by him, and the jealousies of his fellow-workers, another man was elected to the professorship, Carl Heitzmann was disappointed, and with a desire for surroundings less oppressive, and with less deep ruts in which he was expected to run, came to New York toward the end of the year 1874 and established his laboratory.

It may be stated here that Carl Heitzmann made most of his discoveries while working in the laboratory of Stricker Professor of General Pathology in the University of Vienna, and had not Stricker worked against him and refused to accept his discoveries as true, Heitzmann would have had the professorship he wanted. However, in 1880, Stricker published the results of researches of his own, principally regarding the cornea, and accepted fully Heitzmann's claims of six years previous. The English physiologist, Drysdale, in 1865, compared the cell to a gun barrel without lock or stock—so imperfect did the theory seem to him, and this was after Lionel Beale and Max Schultze had established their protoplasma theory, which they tried to conform to the cell theory. Heitzmann felt the same dissatisfaction with the so-called cell doctrine, though with greater force, and resolved to prove it true or to disprove it if it was, as he thought, false.

Upon Schwann's investigation of the constitution of cartilage, his cell doctrine was to a great extent based. Heitzmann's bioplasson doctrine was formed from what he saw in his examinations of the same tissue, though it would seem to be the least suited of all tissues for the demonstration of his peculiar claims, on account of the large proportion of basis substance. Heitzmann found the formerly supposed non-connecting cartilage cells to be mases of living matter, the nuclei and granules of which were connected by . fine lines of a protoplasmic nature, making a network within and that each corpuscle the corpuscle was connected with all others in the vicinity by means of—to use the appropriate term suggested by Prof. L. Elsberg of New York—a bioplasson reticulum, which pierced the gule-yielding basis-substance in all directions. Dr. Heitzmann has demonstrated that the formerly so called protoplasm of tissue in general is not, as was supposed, stuctureless, but that it has a reticular structure, the reticulum being the living matter proper, on the contraction and extention of which depends the change of the shape and locomotion in the simplest animal organism, in the highest organs of man. Not only is there a connection by means of the reticulum between all portions of living matter in the same tissue, but the bioplasson of each tissue is intimately connected, by fine off-shoots, with that of the tissue lying contiguous to it. This body of ours, then, in place of being made up of colonies of structureless amœba, as claimed by Hæckel and Huxley, may be said to resemble an enormous amœba, with a bioplasson reticulum absolutely literally connected throughout, enclosing in its meshes of the accompanying basis-substance enclose a glue-giving material, which is the lifeless fluid above referred to, chemically changed and solidified. Isolated lumps of living matter are found only suspended in the liquids, blood and lymph, and in the different.

Two year ago A. Spine, of Vienna, treating cartilage with alcohol, discovered a ready and simple means for demonstrating the connection of the cartilage corpuscles—so simple, indeed that to-day an eye little experienced, and with comparatively low power, con see what in 1873 Carl Heitzmann maintained, based upon rather tedious method of staining the cartilage with nitrate of silver and chloride of gold. Carl Heitzmann's laboratory comprises the entire fourth floor of his residence and is a convenient and well-lighted apartment. The number of work tables, at first four, is now seventeen, this large increase having been necessitated by the rapid filling up of the classes, over 700 gentlemen having studied there since the laboratory was established.

The study of the minute anatomy of the teeth has made remarkable progress during the last four years, and principally through the efforts of Carl Heitzmann and a few co-workers, among whom is Dr. C. F. W. Bödecker, of New York, who has contributed the most knowledge to the departments included in this section. It may be well to refer briefly to a few of the most important discoveries by Dr. Bödecker and others concerning the structure and relative arrangement of the organic and inorganic constituents of the dental organ—based, all of them, as far as possible, upon C. Heitzmann's bioplasson doctrine.

Much advantage was gained by these gentlemen in not confining their examinations to specimens cut from the dried tooth—it being easily seen that in the progress of drying, the soft tissues would shrink, lose their form and occupy much less space than during life. Then, too, dried specimens have to be round; consequently, the syaces (lacunæ, tubuli, etc.) would collect more or less dirt, which would confuse the examiner, dirt not always having its name upon its face so that it may be recognized as such. Fresh speci mens were cut with the razor from teeth which had been immersed in a one-half to one per cent. solution of chromic acid.

It is not necessary to go into the late methods of preparing specimens, which are many—all tending toward the preservation of the form and natural position of the soft tissues and the prevention of the accumulation of foreign substances. The dentinal tubuli radiate from the boundary of the pulp canal, differing in curvature according as they are directed toward crown, neck or root of the tooth. Each tubule or canaliculus runs in a wavy course, branching usually only as it approaches enamel or cementum, and contains a slightly beaded fibre which is smaller than the calibre of the canaliculus. From the periphery of these fibres fine off-shoots may be seen, with high power, which point toward corresponding opening in the walls of the canalicali, though they are often so fine as to prevent the examiner from really seeing their exit through these little opening; but in stained specimens a fine reticulum of bio-plasson can plainly be seen piercing the basis-substance between the canaliculi, and its connection with the dentinal fibres is shown at many if not all points. The canaliculi and their contents branch and ramify in an exaggerated manner as they approach the enamel and cementum, the increased sensitiveness of dentine at these points during dental operations being accounted for by the presence of a lager proportion of living matter. The dentinal fibres are connected with the odontoblasts of the pulp in the growing tooth and with its protoplasmic bodies in the adult tooth, no regular odontoblasts being present. The periphery of the dentine next the cementum does not contain canaliculi, a finely granular layer varying in thickness being found here, the bay-like excavation seen at the juncture of dentine and cementum often containing protoplasmic bodies possessing off-shoots which serve to connect the branches of the dentinal fibres with those of the cement corpuscles. This layer of protoplasmic bodies between dentine and cementum, as also between dentine and enamel, has been named by Dr. W. H. Atkinson the interzonal layer.

The connection of the dentinal fibers, with the cement corpuscles is also established by the anastomosis of the fibres, or their large branches, with the coarser off-shoots from the protoplasmic bodies of the cementum, and by the fine reticulm of the basis-substance of the dentine passing into that of the cementum. This is the only method of connection at the neck of the tooth, the dentinal reticulum starting from small pear-shaped enlargements on the terminations of the fibres.

The connection can also be seen between the living matter of the cementum and nucleated protoplasmic bodies of the pericementum, excepting at the neck of the tooth, where the cementum is covered by epithelial elements which turn over into the epithelial coating of the gum, and closely resembling those comprising the so-called Nasmyth's membrane which covers the enamel of the young tooth.

Tomes describes the human enamel as a structure containing but 3.5 per cent. of organic matter, the remaining 96.5 per cent. being lime-salts. He tells us of the enamel rods, has much to say about their shape, their wavy course, etc., and then adds: "The rods are solid, are in absolute contact with one another and have no demonstrable intervening or uniting substance."

Dr. Bödecker also admits the existence of the enamel rods and agrees with Tomes in regard to their course, etc., but he sees and demonstrates to others the existence of what he calls "enamel fibres" of living matter in the interstices between the rods. Branching from these fibers at right angles with them there may be seen minute conical fibrillæ which traverse the space between the fibres and the contiguous rods, enter the rods and are then lost to view, though in the thoroughly decalcified specimens these fibrillæ may be seen traversing the substance of the rods proper, and uniting to form a network similar to the reticulum in the basis-substance of the dentine. Tomes informs us of the existence of organic matter in enamel, but does not tell us in what form or at what points it may be found. Bödecker not only gives us full information in regard to the position of the organic matter, but he prepares specimens and adjusts them so that the novice can see this fine reticulum in the enamel rods.

Dr. Bödecker has also demonstrated the morbid changes which take place in the formation of secondary dentine and in inflammation of the peri-cementum and pulp—all of which has been written up in the able manner characteristic of him, and published in the *Cosmos*.

Dr. Frank Abbott has spent much time in the examination of tooth caries, and, though the subject is too formidable to be mastered by the researches of one man, he has certainly added much to our knowledge of that pathological condition. He is at present pursuing studies concerning the changes of the dental tissues during the process of absorption of the teeth of the first dentition.

A very valuable book, and one which we will all want, is soon to be issued from a New York press. It is from the pen of Dr. Heitzmann, is upon general histology and microscopy, and its title is "Microscopic Morphology." It will contain a complete explanation of Dr. Heitzmann's views as well as the researches of a number of its co-workers, including Dr. L. Elsberg of New York, and it will not only be a very interesting work but the most valuable one for the advancement of science which has been published for years.

Every dentist present should take the first opportunity of meeting Dr. Heitzman. He is a large-hearted, genial gentleman, very courteous, and, of course, very enthusiastic upon the subject of microscopy. His aim is, he says, to raise the standard of dentists in this country in their strictly scientific attainments, and I am sure he has already done much for the profession in that direction.

His views, although nearly revolutionary in comparison with those held by previous histologists, are not like a sieve —they will hold water—and I defy the greatest unbeliever not to be converted to the bioplasson doctrine after looking at Dr. Heitzmann's specimens through the microscope and listening to one short course of his lectures.

This report cannot close without referring to the great friendship existing between Dr. Heitzmann and our own dear Dr. Atkinson, or "Papa" as Dr. Heitzmann delights to call him. When Carl Heitzmann first came to this country, Dr. Atkinson, becoming convinced of the truth of the bioplasson doctrine, did much to introduce him, and assisted in making the laboratory the success which it soon became; and to the cordial support given by Drs. Atkinson, Mills, Bödecker, Abbott, and a few other prominent dentists, to

Dr. Hetizmann's views, is due his great interest in the profession at large, and his desire to add to our knowledge of the departments of Section 1.—*New England Journal of Dentistry.*

